# Impact of Bedside Echocardiography in the Management of Critically Ill Pediatric Patients in the Pediatric Intensive Care Unit: A Cross-Sectional Study

**DOI:** 10.7759/cureus.69718

**Published:** 2024-09-19

**Authors:** Sri Sita Naga Sai Priya K, Amar Taksande

**Affiliations:** 1 Pediatrics, Jawaharlal Nehru Medical College, Datta Meghe Institute of Higher Education and Research, Wardha, IND

**Keywords:** critically ill pediatric patients, echocardiography, outcome, pediatric intensive care unit, ventricular septal defect

## Abstract

Background

Managing critically ill pediatric patients is a challenging responsibility that necessitates effective prioritization and time management. It is important not only to assess the condition of the patient on a continuous and real-time basis but also to assess in a way that will provide vital clues that may help in diagnosis and treatment. Our study aims to investigate the association between echocardiography and clinical systemic examination, to find the association between cardiac dysfunction and pediatric outcomes, and to identify the indications and necessity of echocardiography assessments and therapeutic interventions for patients in the pediatric intensive care unit.

Methods

This cross-sectional study was conducted in the pediatric intensive care unit (PICU) of the pediatrics department at Datta Meghe Institute of Higher Education and Research, Wardha, India. All critically ill pediatric patients admitted to the PICU underwent echocardiography. The study primarily focused on the indications for echocardiography, the association between systolic and diastolic dysfunction and patient outcomes, and the therapeutic interventions implemented based on the echocardiographic findings.

Results

The study analyzed 139 subjects aged from one month to 204 months, with the majority in the one- to five-year age group, followed by the 10-15-year age group. Cardiac anomalies were identified in 39 out of 139 cases, and cardiac murmurs were present in 27 cases with an almost equal gender distribution in the infantile age group. Dyspnea, edema, and hepatomegaly were the most common indications for echocardiography. Lasix was the most commonly used antifailure drug used in heart diseases. Sixteen individuals had systolic dysfunction, and eight had diastolic dysfunction, with a mortality rate of 62%. There was a significant association between systolic and diastolic dysfunction and mortality.

Conclusion

Echocardiography is a valuable asset within the PICU, providing critical insights into cardiac function and hemodynamics. By guiding clinical decision-making, it plays a pivotal role in optimizing care strategies, ultimately leading to improved outcomes for pediatric patients admitted with cardiac conditions. Overall, the study emphasizes the complex nature of pediatric cardiac conditions and the necessity for individualized treatment approaches based on distinct diagnoses and clinical indications.

## Introduction

Managing critically ill pediatric patients is a demanding task that requires meticulous prioritization and efficient time management. The clinical picture is often complicated by overlapping symptoms due to multi-system involvement. In recent times, hemodynamic assessment has gained prominence in the treatment of critically ill patients, reflected by the shift from the Airway, Breathing, Circulation (ABC) approach to the Circulation, Airway, Breathing (CAB) approach [1]. Echocardiography is now crucial to clinical care in pediatric intensive care units (PICUs). It is a practical bedside imaging tool and an effective diagnostic method for assessing critical body systems. By combining echocardiographic results with clinical observations, healthcare providers can thoroughly understand the hemodynamic status of critically ill children. This study highlights the clinical uses of echocardiography within the PICU setting. Echocardiography is regarded as a practical and non-invasive bedside imaging technique, offering precise diagnostics for evaluating critical body systems [2]. One of its significant advantages is being a non-invasive procedure that can be performed repeatedly and in real-time. Pediatric intensivists utilize echocardiography alongside clinical information to guide management decisions. Research indicates that echocardiography significantly influences treatment and alters the disease trajectory in 30% to 60% of critically ill patients following its use [3].

Research suggests that PICUs will likely adopt echocardiography as a standard practice in the future [3]. It is advantageous for bedside clinicians to have proficiency in basic echocardiographic assessments, as these evaluations can impact patient treatment, even though the most valuable parameters are still being determined. This study aims to identify the indications for echocardiographic assessments in critically ill children, investigate the relationship between systolic and diastolic dysfunction and patient outcomes, and correlate echocardiographic findings with clinical systemic examinations of PICU patients.

## Materials and methods

Study design, setting, and study period

This prospective, cross-sectional study involved all the critically ill pediatric patients admitted to the PICU. It was carried out over two years in the PICU within the Department of Paediatrics at Acharya Vinoba Bhave Rural Hospital (AVBRH) in Sawangi, Wardha, India.

Definition of Critically Ill Pediatric Patient

The study involves high-risk pediatric patients who are experiencing severe issues related to airway, breathing, or circulation, as well as acute consciousness deterioration. This includes conditions such as apnea, upper airway obstruction, hypoxemia, central cyanosis, severe respiratory distress, complete feeding inability, shock, severe dehydration, active bleeding requiring transfusion, unconsciousness, or seizures.

Study criteria (inclusion and exclusion)

Inclusion criteria consisted of critically ill pediatric patients (one month to 18 years of age) admitted to the PICU. Exclusion criteria excluded postoperative patients.

Ethics consideration and sample size

The Institutional Review Board of Datta Meghe Institute of Higher Education and Research, Wardha, approved the study protocol under the reference number DMIMS(DU)/IEC/2022/1073. 

The formula used for the calculation of sample size is as follows: n = [DEFF*Np(1-p)] / [(d2/Z21-α/2*(N-1)+p*(1-p)] 

Study procedure

**Figure 1:**
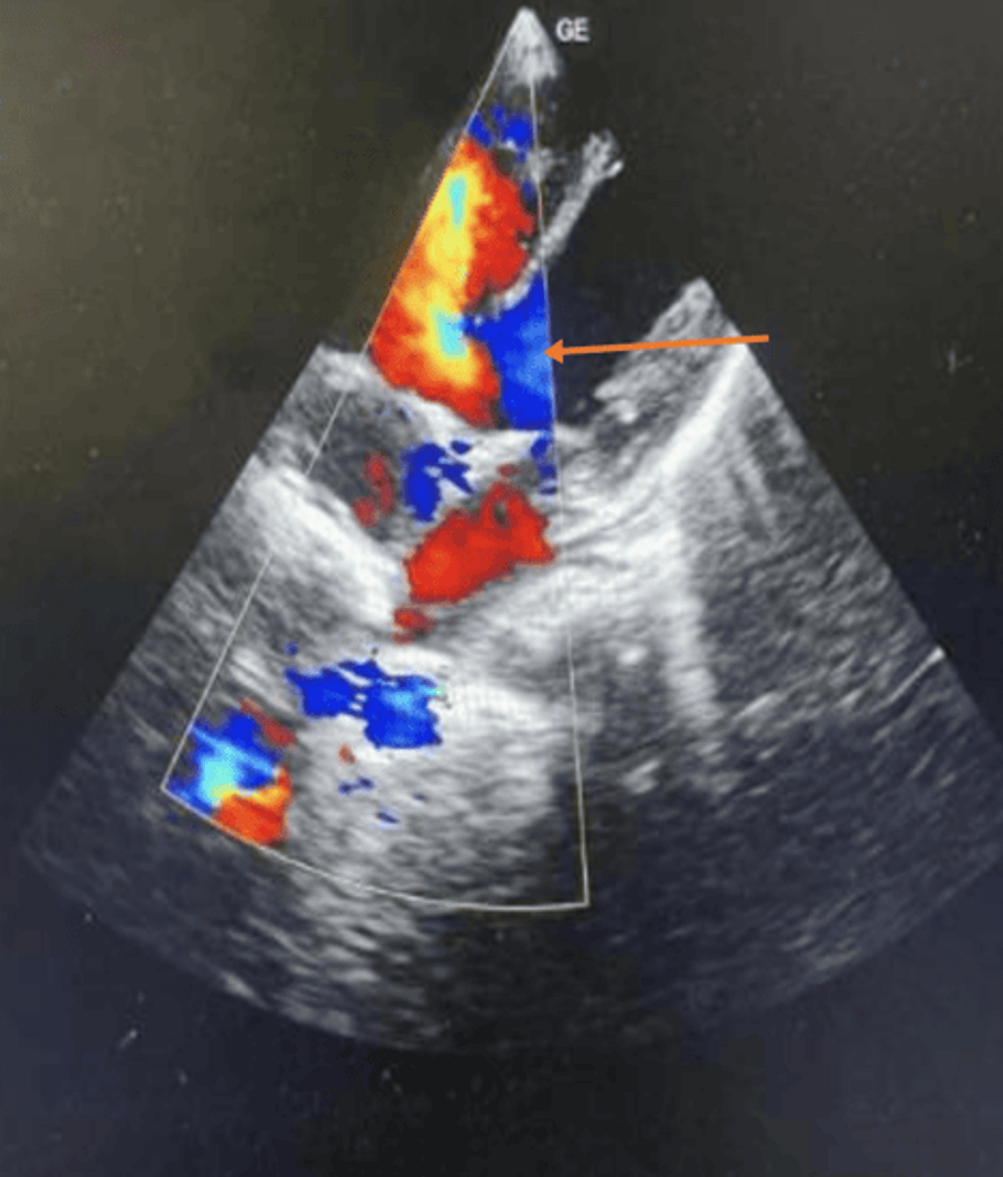
Echocardiography shows a large sub-aortic ventricular septal defect (VSD) in four chamber view.

Patients were classified into subgroups based on diagnosis, including age, gender, primary illness, and type of mechanical ventilation (non-invasive or invasive). Echocardiography indications included symptoms suggesting congestive heart failure, arrhythmias, changes in systemic pressures, and varying medical conditions that required cardiac evaluation. Bedside echocardiography was conducted using a portable ultrasound machine, with standard views and measurements taken to assess cardiac function and fluid status and detect structural abnormalities. The results were analyzed, noted, and compared with other echocardiographic findings. Figure 1 shows echocardiography of the subaortic ventricular septal defect in four-chamber view.

**Figure 2:**
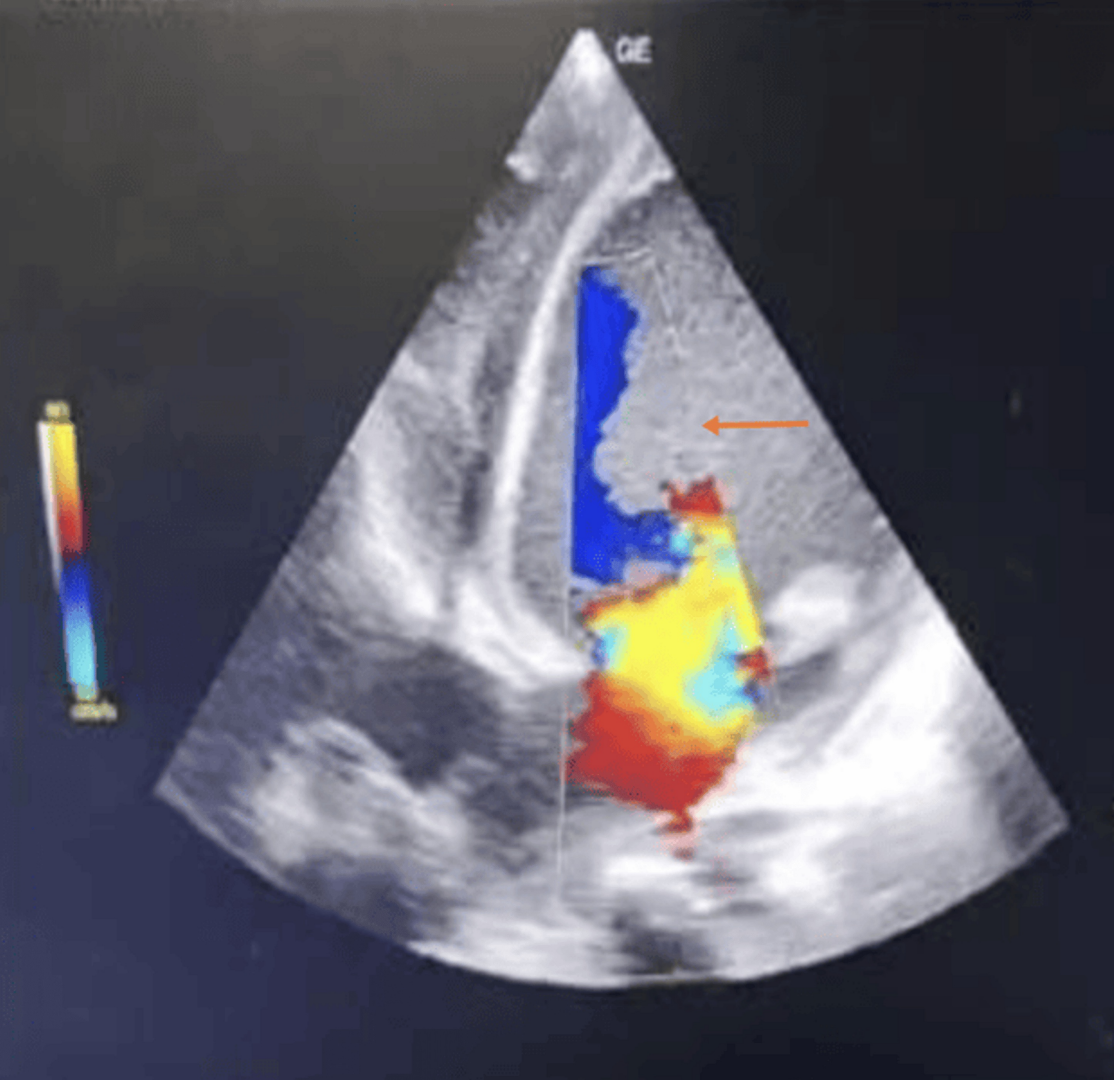
Echocardiography shows left ventricular dilatation with mitral regurgitation in a case of dilated cardiomyopathy

**Figure 3:**
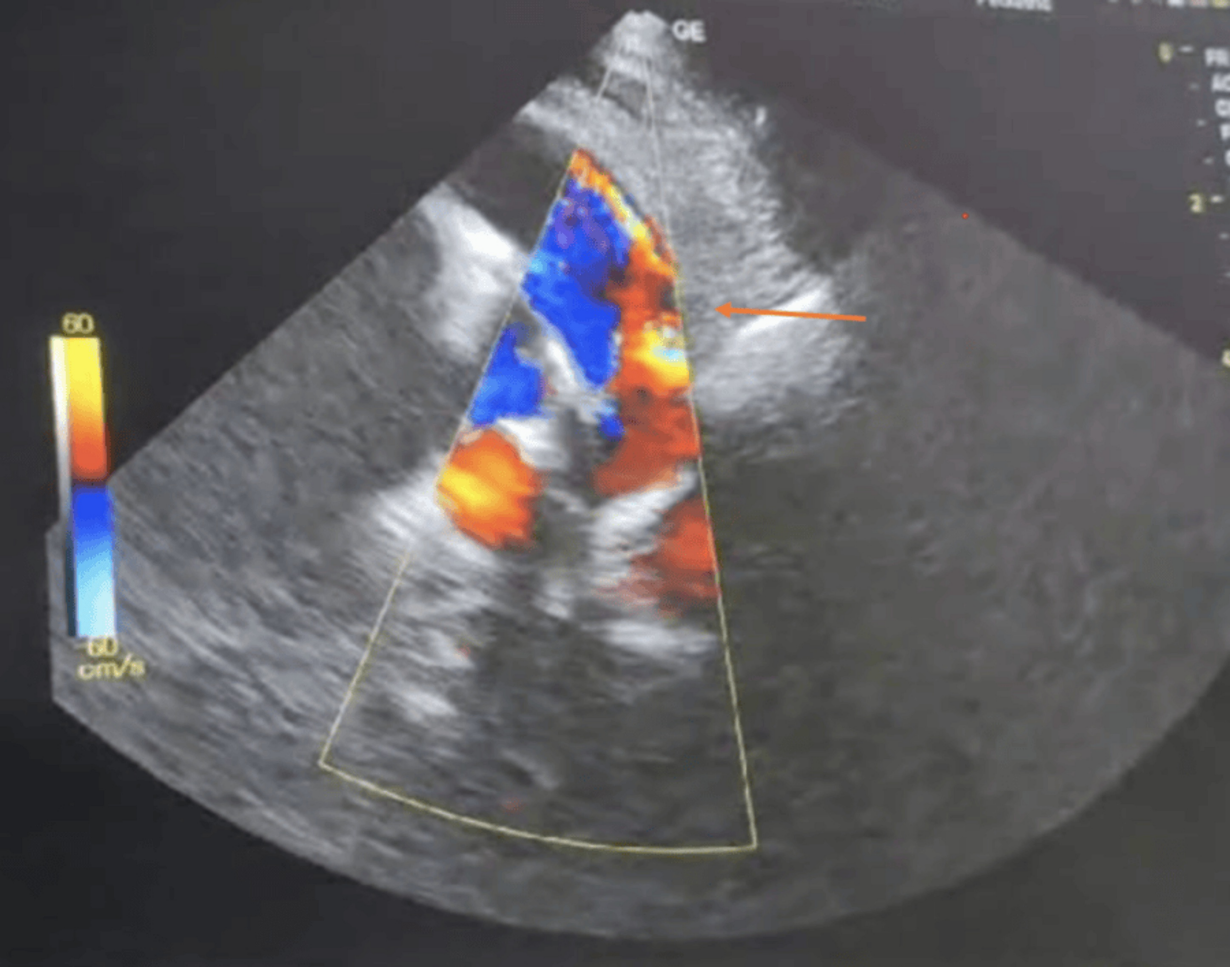
Echocardiography shows patent ductus arteriosus (PDA) in the parasternal short axis view

The procedure involved positioning the child correctly and placing the transducer on the chest to capture specific heart views, such as the apical four-chamber, parasternal short axis, parasternal long axis, and subcostal views. Color Doppler was utilized to evaluate blood flow and detect cardiac anomalies, with imaging settings adjusted for optimal quality. The assessment also included evaluating inferior vena cava (IVC) collapsibility in patients with hypotension and shock. A systemic examination was conducted before the echocardiography. Figure 2 shows echocardiography of left ventricular dilatation with mitral regurgitation in case of dilated cardiomyopathy. Figure 3 shows patent ductus arteriosus on parasternal short axis view. Findings were shared with the healthcare team, and post-assessment care involved removing the probe and gently cleaning the child’s skin for comfort. The entire procedure followed institutional protocols and guidelines for pediatric echocardiography. 

Statistical analysis

Data collection for the study was conducted using Microsoft Excel (Microsoft Corp., Redmond, WA), and statistical analysis involved both descriptive and inferential methods using Stata software (STATA 10, StataCorp LLC, College Station, TX). Quantitative data were analyzed by calculating means, medians, and standard deviations, while qualitative data were summarized using percentages and proportions. The chi-square test and Fisher's exact test were employed to compare differences in proportions. The unpaired Student's t-test was used to compare means, with a significance level set at a p-value of less than 0.05. Descriptive statistics provided an overview of central tendencies and variability, and the Fisher's exact test was applied for small sample sizes to determine significant associations between categorical variables.

## Results

A total of 139 echocardiographies were performed from June 2022 to June 2024 on critically ill patients who met the inclusion criteria. Among these patients were 81 males and 58 females, the majority being between one and five years old. Out of these cases, 92 were discharged, and 35 resulted in death.

Table 1 illustrates the indications for echocardiography in patients with cardiac anomalies. Among these, 27 cases involved cardiac anomalies with murmurs/suspected cardiac disease, followed by six cases presenting with dyspnea, edema, and hepatomegaly. In total, 39 individuals had either structural or functional cardiac anomalies.

**Table 1:** Indications of echocardiography with cardiac anomaly The data have been represented as N(%)

Indication	Cardiac anomaly	
	Present N(%)	Absent N(%)
Murmurs, suspected cardiac disease	27 (100)	0 (0)
Hypotension, shock	5 (14.7)	29 (85.2)
Hypertension	0 (0)	10 (100)
Arrhythmia	0 (0)	2 (100)
Dyspnea, edema, hepatomegaly	6 (15.7)	32 (84.2)
Convulsions	0 (0)	20 (100)
Pleural effusion	1 (25)	3 (75)
Acidosis	0 (0)	1 (100)
Hypoxia	0 (0)	1 (100)
Intracerebral hemorrhage	0 (0)	2 (100)

Table 2 shows that echocardiography was conducted in 27% of cases with dyspnea, edema, and hepatomegaly, and 24% were for patients with hypotension and shock.

**Table 2:** Indications of echocardiography among study population The data have been represented as N(%)

Indications	Frequency (N(%))
Murmurs, suspected cardiac disease	27 (19.42)
Hypotension, shock	34 (24.46)
Hypertension	10 (7.19)
Arrhythmia	2 (1.44)
Dyspnea, edema, hepatomegaly	38 (27.34)
Convulsions	20(14.39)
Pleural effusion	4 (2.88)
Acidosis	1 (0.72)
Hypoxia	1 (0.72)
Intracerebral hemorrhage	2 (1.44)

Table 3 details the association of echocardiographic diagnosis with gender and murmurs. Among patients with ventricular septal defect, a clinical murmur was detected in seven female and two male cases. 

**Table 3:** Association of echocardiographic diagnosis with gender and murmur ASD: atrial septal defect; VSD: ventricular septal defect. PDA: patent ductus arteriosus; TOF: tetralogy of Fallot; TAPVC: total anomalous pulmonary venous connection; DORV: double outlet right ventricle; RHD: rheumatic heart disease; MR: mitral regurgitation; MS: mitral stenosis.

Echocardiography diagnosis	Murmur				
	Present		Absent		
	Male (n=7)	Female (n=11)	Male (n=74)	Female (n=47)	Total (n=139)
ASD	0 (0.0)	0 (0)	5 (71.43)	2 (28.57)	7(100)
VSD	2 (20)	7 (70)	1 (10)	0 (0)	10(100)
PDA	0 (0)	1 (33.33)	1 (33.33)	1 (33.33)	3(100)
ASD, VSD	1 (100)	0 (0)	0 (0)	0 (0)	1(100)
VSD, PDA	0 (0)	1 (33.33)	1 (33.33)	1 (33.33)	3(100)
Hypoplastic left ventricle	1 (100)	0 (0)	0 (0)	0 (0)	1(100)
TOF	2 (100)	0 (0)	0 (0)	0 (0)	2(100)
Truncus arteriosus	0 (0)	0 (0)	1 (100)	0 (0)	1(100)
Cardiomyopathy	0 (0)	0 (0)	2 (50)	2 (50)	4(100)
TAPVC	0 (0)	0 (0)	2 (100)	0 (0)	2(100)
Tricuspid atresia	0 (0)	0 (0)	1 (100)	0 (0)	1(100)
DORV with complex heart disease	1 (100)	0 (0)	0 (0)	0 (0)	1(100)
RHD with MR	0 (0)	1 (100)	0 (0)	0 (0)	1(100)
Coarctation of aorta	0 (0.0)	0 (0)	0 (0)	1 (100)	1(100)
RHD with MS	0 (0)	1 (100)	0 (0)	0 (0)	1(100)
Normal	0 (0)	0 (0)	60 (60)	40 (40)	100(100)

Table 4 shows the relationship between the indications for echocardiography and the types of medical interventions administered. Patients with murmurs and suspected cardiac disease were primarily treated with Lasix, which reduces preload, accounting for 33.33% of cases. This was followed by the use of inotropes in 25.9% of cases. Other treatments included a combination of inotropes (18.5% of cases) and drugs such as Lasix and enalapril, which reduce preload and after-load respectively (14.8%), and propranolol, which was used in 7.4% of cases.

**Table 4:** Association of indications with the type of medical intervention The data have been represented as N(%)

Indication	No intervention (n=101)	Lasix (n=12)	Lasix and enalapril (n=5)	Lasix, enalapril, and ionotropes (n=9)	Ionotropes (n=10)	Propranolol (n=2)	Total (n=139)
Murmurs, suspected cardiac disease	0(0)	9 (33.33)	4 (14.81)	5 (18.52)	7 (25.93)	2 (7.41)	27(100)
Hypotension, shock	29 (85.29)	0 (0)	0 (0)	2 (5.88)	3 (8.82)	0 (0)	34(100)
Hypertension	10 (100)	0 (0)	0 (0)	0 (0)	0 (0)	0 (0)	10(100)
Arrhythmia	2 (100)	0 (0)	0 (0)	0 (0)	0 (0)	0 (0)	2(100)
Dyspnea, edema, hepatomegaly	33 (86.84)	3 (7.89)	1 (2.63)	1 (2.63)	0 (0)	0 (0)	38(100)
Convulsions	20 (100)	0 (0)	0 (0)	0 (0)	0 (0)	0 (0)	20(100)
Pleural effusion	3 (75)	0 (0)	0 (0)	1 (25)	0 (0)	0 (0)	4(100)
Acidosis	1 (100)	0 (0)	0 (0)	0 (0)	0 (0)	0 (0)	1(100)
Hypoxia	1 (100)	0 (0)	0 (0)	0 (0)	0 (0)	0 (0)	1(100)
Intracerebral hemorrhage	2 (100)	0 (0)	0 (0)	0 (0)	0 (0)	0 (0)	2(100)

Table 5 indicates the association between systolic and diastolic dysfunction and patient outcomes, showing that both types of dysfunction significantly contributed to mortality. There is a positive correlation between systolic and diastolic dysfunction and mortality, with a p-value of 0.0012 and 0.0391, respectively.

**Table 5:** Association of systolic and diastolic dysfunction with outcomes The p-value for systolic dysfunction is 0.0012, the p-value for diastolic dysfunction is 0.0391 DAMA: discharged against medical advice The data have been represented as N(%)

Outcome	Systolic dysfunction		Diastolic dysfunction	
	Present N(%)	Absent N(%)	Present N(%)	Absent N(%)
Discharge	5 (31.2)	87 (70.7)	3 (37.5)	89 (67.9)
Death	10 (62.5)	25 (20.3)	5 (62.5)	30 (22.9)
DAMA	1 (6.2)	11 (8.9)	0	12 (9.2)

## Discussion

The function of echocardiography within the PICU has evolved in recent years. Echocardiography is considered a primary hemodynamic assessment in critically ill patients in the PICU. It is a simple but operator-dependent bedside non-invasive monitoring device that details the patient's cardiac structure, function, and hemodynamic status. It significantly impacts clinical decision-making for further management of PICU patients [4].

The purpose of echocardiography is to estimate cardiac function and to evaluate factors affecting the outcome. It provides helpful information, including size, shape, pumping capacity, assessment of cardiac valves, location, and extent of any tissue damage, if any. This hemodynamic assessment provides physicians with crucial information to promptly guide therapeutic interventions such as initiating, discontinuing, or altering therapy and timely referrals to specialists if cardiac or surgical intervention is necessary [5].

Table 6 shows various studies showing the most common indication of echocardiography. Bhadane [6] identified pulmonary hypertension secondary to lung disease as the most common indication. Abd EI Massih et al. [7] focused on assessing cardiac function, reflecting the broad application of echocardiography in evaluating myocardial performance and identifying structural abnormalities. Ali et al. [8] highlighted known congenital heart disease as a primary indication. Kutty et al. [9] emphasized its use in cases of respiratory distress, suggesting its value in assessing cardiac function in patients presenting with respiratory compromise. AI-Ghwass et al. [10] prioritized evaluating left ventricular function. Rato [11] et al. underscored its importance in evaluating intravascular volume status, highlighting its ability to assess fluid status and guide fluid management in critically ill patients. Spurney et al. [12] identified pericardial effusion as a critical indication, demonstrating echocardiography's capacity to detect and characterize fluid collections around the heart. In the present study, dyspnea, edema, and hepatomegaly were the most common indications, indicating the wide-ranging clinical scenarios where echocardiography is employed to assess cardiac structure and function in patients presenting with these symptoms.

**Table 6:** Studies on the most common indication for echocardiography

Study	Year of publication	Most common indication for echocardiography
Bhadane et al. [6]	2023	Pulmonary hypertension secondary to lung disease
Abd EI Massih et al. [7]	2021	Assessment of cardiac function
Ali et al. [8]	2019	Known congenital heart disease
Kutty et al. [9]	2014	Respiratory distress
AI-Ghwass et al. [10]	2024	Left ventricle function assessment
Rato et al. [11]	2019	Evaluation of intravascular volume status
Spurney et al. [12]	2005	Pericardial effusion
Present study	2024	Dyspnea, edema, hepatomegaly

Table 7 lists that studies regarding the distribution of cardiac anomalies in PICUs have consistently highlighted ventricular septal defect as one of the most common cardiac anomalies. Gundogdu et al. [13], Stoll et al. [14], Reller et al. [15], Marelli et al. [16], Yeh et al. [17], and the present study all identified ventricular septal defect as the most prevalent cardiac anomaly in their respective cohorts. Nale et al. [18] had a bicuspid aortic valve with the most common cardiac anomaly. This consistency across multiple studies underscores the significant clinical impact of ventricular septal defects in pediatric cardiac patients requiring intensive care.

**Table 7:** Studies regarding distribution of cardiac anomalies in PICU VSD: ventricular septal defect; PICU: pediatric intensive care unit

Study	Year of publication	Most common cardiac anomaly
Gundogdu et al. [13]	2019	VSD
Stoll et al. [14]	2015	Septal VSD
Reller et al. [15]	2008	VSD
Marelli et al. [16]	2007	VSD
Yeh et al. [17]	2013	VSD
Nale et al. [18]	2020	Bicuspid aortic valve
Present study	2024	VSD

For patients with a routine echocardiography diagnosis, the majority (100%) did not require any intervention. In cases of atrial septal defect, interventions varied, with single anti-failure (Lasix) and ionotropes each accounting for 28.57% of cases. Among patients with ventricular septal defect, the most common intervention was single anti-failure (Lasix), utilized in 60.0% of cases. In patent ductus arteriosus cases, the management primarily involved either a single anti-failure (Lasix) or a combination of anti-failure and inotropes. Lasix was our present study's most-used anti-failure drug, followed by inotropes used in cardiomyopathy. Masarone et al. [19] concluded that diuretics and angiotensin-converting enzyme inhibitors are the primary treatments. At the same time, beta-blockers and electrical therapy devices are less commonly used in children than adults. For end-stage disease, heart transplantation is the optimal treatment option. 

Moffett et al. [20] reported that diuretics were prescribed in 90.1% of the population, with furosemide being the most recommended. Despite observing a decrease in intravenous furosemide usage over time, from 80.2% in 2001 to 64.1% in 2010, this decline was not deemed statistically significant. The impact of a mechanical ventilation type on systolic and diastolic blood pressure was notable. In our study, patients under non-invasive ventilation exhibited higher mean systolic and diastolic blood pressures than those on mechanical ventilation. It's well documented that mechanical ventilation can trigger cardiovascular and blood pressure fluctuations, as it influences cyclic changes in the aortic blood flow, vena cava, and pulmonary artery. These effects are reflected in blood pressure swings due to patients' volume status [21]. Additionally, the degree of positive end-expiratory pressure application, although not impairing right ventricular hemodynamics at lower values, may reduce cardiac output at higher levels in select patients [22].

Our study revealed that the mean systolic and diastolic blood pressure were lower during invasive types of ventilation, such as synchronized intermittent mandatory ventilation (SIMV) or pressure control ventilation (PCV), in contrast with non-invasive ventilation modalities. After most patients were discharged, we compared the outcomes among systolic and diastolic dysfunction patients. Cardiac dysfunction can manifest as systolic or diastolic, depending on the nature of the cardiac lesion. Left ventricular diastolic function determines left ventricular filling and stroke volume. Abnormalities in diastolic function are common in various cardiovascular diseases and correlate with poorer outcomes, such as increased mortality and heart failure-related hospitalizations. Echocardiography allows for diagnosing diastolic dysfunction and understanding the underlying pathophysiologic mechanisms, which impact the structure and function of the left ventricle and left atrium. It is characterized by the ventricle's ability to fill adequately even when atrial pressure is expected [23].

Levene et al. [24] state that although systolic dysfunction presents a higher overall risk of death and hospitalizations due to cardiovascular issues or heart failure compared to diastolic dysfunction, the risk of cardiac arrest in patients with grade II and III diastolic dysfunction is similar to those with moderate and severe systolic dysfunction, respectively. Therefore, diastolic dysfunction indicates a significant risk of both mortality and morbidity. In the study by Liu et al. [25], severe diastolic dysfunction identified through echocardiography is independently linked to higher all-cause mortality in patients with mid-range or reduced ejection fraction heart failure.

Our study discovered a notable correlation between systolic and diastolic dysfunction and mortality, with a significant p-value of 0.0012 and 0.039. The diverse echocardiographic diagnosis and corresponding interventions outlined in the study highlight the necessity of personalized treatment approaches based on specific cardiodiagnosis and clinical indications. This customized approach is crucial for optimizing patient care, improving treatment outcomes, and minimizing potential complications.

Limitations

This study has several limitations that may impact the accuracy and generalizability of the findings. Firstly, long-term complications could not be assessed due to the lack of follow-up, which limits the ability to evaluate the sustained effectiveness and safety of the interventions. Additionally, echocardiography images were affected by factors such as patient movement, poor acoustic windows, and artifacts, potentially compromising the accuracy of the results. In critically ill patients, who often present with multi-organ dysfunction, echocardiography primarily focuses on cardiac function and may not fully assess other crucial aspects of the patient's condition, such as pulmonary status and neurological function. Moreover, the quality and interpretation of echocardiographic images are heavily influenced by the skill and experience of the operator, introducing variability that can be particularly problematic in a critical care setting where timely and accurate information is essential. This reliance on operator proficiency represents a significant limitation of the study.

## Conclusions

In conclusion, this cross-sectional study highlights the significant impact of bedside echocardiography in the management of critically ill pediatric patients within the PICU. The ability to perform real-time, non-invasive cardiac assessments at the bedside has proven to be a valuable tool in the prompt diagnosis and treatment of hemodynamic instability, guiding therapeutic decisions, and improving patient outcomes. Bedside echocardiography facilitates timely interventions, reducing the need for more invasive procedures and minimizing delays associated with patient transfer to imaging facilities. The findings underscore the importance of integrating bedside echocardiography into routine PICU practice, supporting its role as an essential component in the comprehensive management of critically ill pediatric patients. Further research is warranted to explore its long-term benefits and potential to standardize its use across various clinical settings.
